# Enhanced musical rhythmic perception in Turkish early and late learners of German

**DOI:** 10.3389/fpsyg.2013.00645

**Published:** 2013-09-20

**Authors:** M. Paula Roncaglia-Denissen, Maren Schmidt-Kassow, Angela Heine, Peter Vuust, Sonja A. Kotz

**Affiliations:** ^1^Department of Neuropsychology, Max Planck Institute for Human Cognitive and Brain SciencesLeipzig, Germany; ^2^Institute of Medical Psychology, Goethe University FrankfurtFrankfurt am Main, Germany; ^3^Department of Psychology, Freie Universität BerlinBerlin, Germany; ^4^Department of Clinical Medicine - Center for Functionally Integrative Neuroscience, Aarhus UniversityAarhus, Denmark; ^5^The Royal Academy of MusicAarhus/Aalborg, Denmark; ^6^School of Psychological Sciences, The University of ManchesterManchester, UK

**Keywords:** speech rhythm, L2, musical rhythm, rhythmic aptitude, Turkish, German, English

## Abstract

As language rhythm relies partly on general acoustic properties, such as intensity and duration, mastering two languages with distinct rhythmic properties (i.e., stress position) may enhance musical rhythm perception. We investigated whether competence in a second language (L2) with different rhythmic properties than a L1 affects musical rhythm aptitude. Turkish early (TELG) and late learners (TLLG) of German were compared to German late L2 learners of English (GLE) regarding their musical rhythmic aptitude. While Turkish and German present distinct linguistic rhythm and metric properties, German and English are rather similar in this regard. To account for inter-individual differences, we measured participants' short-term and working memory (WM) capacity, melodic aptitude, and time they spent listening to music. Both groups of Turkish L2 learners of German perceived rhythmic variations significantly better than German L2 learners of English. No differences were found between early and late learners' performance. Our findings suggest that mastering two languages with different rhythmic properties enhances musical rhythm perception, providing further evidence of shared cognitive resources between language and music.

## Introduction

Over the last few decades the impact of bilingualism and second language learning (L2) on cognitive processes has been the objective of many studies. Previous research reported a positive association between bilingualism and verbal and non-verbal intelligence (Peal and Lambert, 1962), problem-solving skills (Bialystok, 1999; Bialystok and Shapero, 2005), phonological memory (Service, 1992; Cheung, 1996), and working memory (WM) capacity in attention-impeding tasks (Yang et al., 2005).

Similarly, musical aptitude has been related to enhanced cognitive abilities (Draper and Gayle, 1987; Milovanov et al., 2008), such as general intelligence (Schellenberg, 2004), verbal memory (Brandler and Rammsayer, 2003), and to the enhanced processing of acoustic features embedded in complex musical contexts (Kraus and Chandrasekaran, 2010; Garza Villarreal et al., 2012; Vuust et al., 2012).

More recently, attention has been drawn to the association between musical aptitude and L2 learning (Milovanov and Tervaniemi, 2011). Studies report a positive effect of musical aptitude on second language skills, such as pronunciation (Milovanov et al., 2008) and phonological perception (Slevc and Miyake, 2006). In addition, it has been shown that a second language may enhance musical aptitude with respect to tone perception (Deutsch et al., 2006; Elmer et al., 2011). Nevertheless, as far as this study is concerned, the impact of second language learning on musical rhythm aptitude has not been investigated.

Similarly to rhythm in music, speech rhythm relies on acoustic prominence to create perceptual units that support the structuring and the organizing of speech flow (Lerdahl and Jackendoff, 1983; Nespor and Vogel, 1986; Hayes, 1989; Jackendoff, 1989). These perceptual units may constitute the basis of language rhythmic classifications as stress-timed, syllable-timed, and mora-timed languages (Pike, 1945; Abercrombie, 1967; Ladefoged, 1975). In stress-timed languages, such as German and English, the unit of speech organization is the metric foot, i.e., a stressed syllable dominates at least one relatively weaker syllable (Hayes, 1985; Nespor and Vogel, 1986). In syllable-timed languages, such as Turkish and French, the syllable, regardless of stress, organizes and structures speech (Pike, 1945; Ladefoged, 1975; Cutler, 1994; Grabe and Low, 2002; Nazzi and Ramus, 2003). Finally, in mora-timed languages (e.g., Japanese), the mora, a subunit of the syllable, is regarded as the speech organization unit (Itô, 1989; Otake et al., 1993; Warner and Arai, 2001)^1^.

At the word level, rhythm operates by means of stress assignment, determining a language's metric preference. In terms of their metric preference, languages rely on the trochee or the iamb as their default metric pattern (Hayes, 1985; Hay and Diehl, 2007). The trochee is characterized by one stressed syllable followed by at least one relatively weaker syllable, while the iamb displays the opposite metric pattern, namely at least one unstressed syllable followed by a stressed one (Hayes, 1985). German and English provide examples of trochaic languages, while Turkish and French are iambic (Eisenberg, 1991; Inkelas and Orgun, 2003).

Implicit knowledge and the use of rhythmic properties such as the organization, perceptual units, and metric preference, constitute part of the speaker's competence in a language (Patel, 2008). Therefore, to master a second language, its rhythmic properties must be learned as part of the linguistic inventory of this language.

Despite speech rhythm being a language-specific ability, it is based on acoustic properties, such as intensity and duration that are found in other auditory domains such as music (Lerdahl and Jackendoff, 1983; Patel, 2003, 2008; Tincoff et al., 2005; Bispham, 2006). Properties of speech rhythm can therefore be considered domain-general properties (Jackendoff, 1989; Hay and Diehl, 2007). Mastering two languages with different rhythmic properties may thus enhance the sensitivity to these general acoustic properties when used in a specific language context.

This should be the case as speech rhythm may support language discrimination (Beckman, 1996; Ramus et al., 1999, 2003; Patel, 2008). Thus, if sensitivity to rhythmic properties in speech enhances the perception of rhythmic properties in music, such evidence would support the notion of shared resources in these two domains. It would also suggest that a domain-specific skill may be transferred to another cognitive domain, e.g., music (Perkins and Salomon, 1989).

Furthermore, if mastering languages with different rhythmic properties positively impacts musical rhythm perception, this effect could also be modulated by L2 age of acquisition (AoA). As some studies suggest, L2 learners have to make use of rhythmic information in an L2 to some extent to acquire this language (Goetry and Kolinsky, 2000). In addition, studies reveal that highly proficient late learners are sensitive to L2 rhythmic properties (Goetry and Kolinsky, 2000; Field, 2003; Trofimovich and Baker, 2006). However, one cannot disregard previous findings revealing that early L2 learners make use of rhythmic strategies in their dominant language only to segment words (Cutler et al., 1986, 1992; Otake et al., 1993). This would imply that similarly to phonology (Flege et al., 1999; Piske et al., 2001) the use of rhythmic strategies in speech segmentation would be constrained by the AoA. As contradictory as these findings may appear, the fact that L2 learners superimpose rhythmic segmentation strategies of their dominant language onto an L2 does not exclude the possibility that they are sensitive to general acoustic properties underlying rhythm in both languages.

In the current research, we addressed two main issues. First, we investigated the impact of mastering languages with different rhythmic properties, such as metric preference and rhythmic classification, on musical rhythmic aptitude. This is motivated by their commonalities in temporal organization (rhythm) of music and language. In both domains rhythm organizes acoustic events in terms of timing and grouping, structuring the acoustic input in a hierarchical fashion by means of perceptual units (Lerdahl and Jackendoff, 1983; Nespor and Vogel, 1986; Hayes, 1989; Jackendoff, 1989).

Second, we explored whether musical rhythm can be modulated by L2 AoA. Even though much is known about the impact of AoA on different L2 skills, such as phonology, semantics, and syntax (Johnson and Newport, 1989; Weber-Fox and Neville, 1996; Flege et al., 1999; Piske et al., 2001; Wartenburger et al., 2003; Ojima et al., 2005; Clahsen and Felser, 2006; Hernandez and Li, 2007), the same does not hold true for L2 rhythm (Chun, 2002; Trofimovich and Baker, 2006). It could be that either the attainment of L2 rhythm is constrained by AoA as suggested by some research on rhythmic strategies in word segmentation (Cutler et al., 1986; Otake et al., 1993; Guion et al., 2004), or that it may be acquired with increased L2 exposure and proficiency (Goetry and Kolinsky, 2000; Field, 2003; Trofimovich and Baker, 2006).

In order to address these issues, we tested Turkish early (TELG) and late L2 learners of German (TLLG) and German late L2 learners of English (GLE) with respect to their musical rhythmic aptitude. Whereas German and English share rhythmic classification and metric preferences (Pike, 1945; Eisenberg, 1991; Cummins and Port, 1998), Turkish and German represent rather an interesting contrast when considering their respective rhythmic properties. While German is a stress-timed language with a metric preference for the trochee, Turkish is syllable-timed and uses the iamb as its default metric pattern (Eisenberg, 1991; Grabe and Low, 2002; Inkelas and Orgun, 2003; Nazzi and Ramus, 2003; Topbas, 2006; Höhle et al., 2009).

In order to control for individual differences that may influence participants' performance, such as cognitive and musical ability, participants were tested in terms of their short-term memory (STM) and working memory (WM) capacities. Short-term memory regards the ability to store given, and relatively unprocessed, information for a short period of time (Baddeley, 2003; Conway et al., 2005). Working memory characterizes the ability to maintain information actively while cognitive processes are being executed (Baddeley, 2003; Conway et al., 2005). Previous research suggests that STM and WM capacity correlate with general intelligence, thus providing an indicator of cognitive resources (Daneman and Carpenter, 1980; Engle et al., 1999; Oberauer et al., 2000; Conway et al., 2005; Unsworth and Engle, 2007).

In addition, participants were asked about their musical background, weekly exposure to music, and were tested for their musical aptitude, by means of a melody aptitude test. Next to rhythm, the perception of pitch variation, as in melody and harmony, is considered one of the two fundamental aspects of music (Lerdahl and Jackendoff, 1983) and is extensively used as an indicator of musical aptitude (Seashore et al., 1960; Gordon, 1969, 2007; Wallentin et al., 2010).

Therefore, by controlling for differences in participants' cognitive ability, musical aptitude, and weekly exposure to music, we expected differences in rhythmic aptitude to be explained by the mastery of languages with distinct rhythmic properties.

## Materials and methods

### Participants

Eighty-five right-handed participants were assigned to three experimental groups, i.e., 27 Turkish late L2 learners of German (13 females, *M*_age_ = 29.11, *SD* = 3.85, mean age of L2 first exposure, AoL2FE = 20.03, *SD* = 6.40), 26 Turkish early L2 learners of German (12 females, *M*_age_ = 26.80, *SD* = 4.48, *M*_AoL2FE_ = 1.03, *SD* = 0.19) and 32 German late L2 learners of English (16 females, *M*_age_ = 25.71, *SD* = 2.55, *M*_AoL2FE_ = 10.04, *SD* = 1.27). All participants were non-musicians. 64.7% of the participants reported playing no instrument at all, while 35.3% reported playing an instrument for an average of 4.75 years (*SD* = 3.83). Participants were either university students or recent graduates. They were paid for their participation. None of the participants reported any neurological impairment or hearing deficit, and all had normal or corrected-to-normal vision. This study was approved by the ethics committee of the University of Leipzig and all participants gave their written informed consent for data collection, use, and publication.

### Second language assessment and language history questionnaire

All participants were given a language history questionnaire concerning both their L1 and L2. With this questionnaire, we assessed language competence, such as listening, writing, reading and speaking skills, age of first exposure to the languages, situations in which each language was acquired, and current language use. Self-reported language questionnaires have been successfully used to assess L1 and L2 acquisition, history and competence skills (Elston-Güttler et al., 2005; Marian et al., 2007; Schmidt-Kassow et al., 2011a). Based on the results of the assessment and on the participants' own perception of their language preference, English and German were regarded as the second language among German and in both Turkish L2 learner groups, respectively.

### The musical ear test

As a rhythmic aptitude measure, we used the rhythmic subset of the Musical Ear Test (MET; Wallentin et al., 2010). The MET rhythmic subset consists of 52 rhythmic pairs, which are formed by either two identical or two different rhythmic phrases. All rhythmic phrases were recorded using wood blocks and were 4–11 beats long. Rhythmic phrases have a duration of one measure and were played at 100 bpm. Trials constituted two distinct rhythmic phrases and differed only by one rhythmic change. Rhythmic complexity was achieved by including triplets in 21 trials, while the other 31 trials presented even beat subdivisions. Thirty-seven trials begin on the downbeat while the remaining trials begin on the beat removed. The order, in which these features occurred, was randomized.

In its original version, the MET involves an answer sheet to be filled out by the participants. Additionally, the test provides participants with auditory instructions in English prior to and during the test to introduce each trial. We created an adapted version, in which instructions in German were presented visually prior to the test, i.e., in the training phase, but not before each single trial.

### Short-term memory and working memory measures

In the current study, we used the Mottier Test, MT (Mottier, 1951), a non-word repetition test, as a measure of short-term memory. The MT is composed of sets of 6 non-words, ranging from 2 to 6 syllables each. The stimulus material presented a constant syllabic structure of one consonant followed by one vowel, i.e., CV. The non-words were spoken by a female professional speaker and presented to participants via headphones.

We used the backward digit span (BDS), a WM measure involving information storage and transformation (Oberauer et al., 2000; Süß et al., 2002). The BDS version adopted in the current study is composed of 14 sets of 2 trials, ranging from 2 to 8 numbers. The numbers were spoken by a female German native speaker and recorded at a rate of one number per second. Numbers were presented via headphones and participants had to recall them in the reverse order of which they were presented.

### Melodic aptitude test

To measure participants' melodic aptitude, the melodic subset from the MET was used (Wallentin et al., 2010). This subset consists of 52 melodic pairs, formed by two identical or two different melodic phrases. Melodic phrases consisted of 3–8 tones and had a duration of one measure and were played at 100 bpm. Different trials (26 pairs) contained pitch violation and in half of them the pitch violation also characterized a violation in the pitch contour. Twenty-five trials were constituted by non-diatonic tones, while 7 trials were in the Minor key and 20 in the Major. The order, in which these features occurred, was randomized.

### Procedures

Participants were tested individually in a quiet room. The tests were administered in a pseudo-randomized order on a computer and each individual session lasted ~1 h. Participants received written instructions for each test, either on separate instruction sheets or presented on the computer screen. Before each test, practice trials were provided and participants were allowed to repeat them until the test was understood correctly. At the end of the session, participants were asked about the average time they spent listening to music in a week (number of hours). Furthermore, participants' information about their L1 and L2 was assessed.

#### The musical ear test

The MET rhythmic subset was presented via headphones using a computer. While participants listened to rhythmic phrases, a white star was presented in the center of a black screen, providing a visual cue to attend to during stimulus presentation. Participants judged if the presented rhythmic pair comprised identical or different phrases. At the end of a rhythm trial, the white star was replaced by the words “JA” (*yes*) and “NEIN” (*no*) placed at middle height and at opposite sides of the screen, matching the positions of the response keys. Participants had 1 s to press the corresponding answer key. The position of the correct-response key was counter-balanced across participants.

#### Mottier Test and backward digit span

Participants self-initiated the Mottier Test by pressing the space key. With a visual cue placed in the center of the computer screen, participants heard the first non-word and were instructed to repeat it as accurately and as fast as possible, after which the next non-word was presented and the same procedure was repeated. At the end of each trial set, participants were given a short break and self-determined when the test should be re-initiated. Participants' responses were computed *ad-hoc* by the experimenter with the help of a response sheet, as well as being recorded via the computer. The test was terminated when participants failed to recall a minimum of 4 items of the same trial set correctly. Scoring was based on the total number of correctly recalled non-words.

In the BDS, participants listened to the sequences of numbers via headphones while facing away from the computer. At the end of the numerical trial, participants were asked to repeat the numbers in the reversed order of their presentation. The test was terminated when participants failed to recall two trials of the same set. Scoring was given according to the total number of trials correctly recalled.

#### Melodic aptitude test

The MET melodic subset was presented via headphones using a computer. Participants listened to the melodic phrases while presented with a visual cue in the center of a black screen. Participants were to judge if the presented melodic pair consisted of identical or different phrases. With the end of the melodic trial, participants were presented with the words “JA” (*yes*) and “NEIN” (*no*), matching the positions of the response keys. Participants had 1 s to press the corresponding answer key. Correct-response key position was counter-balanced across participants.

#### Statistical analysis

German late L2 learners of English were divided into three groups according to their self-reported English proficiency level, i.e., having very good to excellent writing and speaking skills, having good writing and speaking skills and having good speaking, but not writing skills in English. An ANOVA was conducted with a between-subjects factor (proficiency) and their rhythmic performance as dependent variable. This allowed to explore whether their knowledge of another language (English) with similar rhythmic properties to German (e.g., Pike, 1945; Jusczyk et al., 1993) would affect their musical rhythmic performance. Furthermore, all participants were divided into three groups, creating a between-subjects factor *group* (German late L2 learners of English, Turkish early and late L2 learners of German). An analysis of covariance (ANCOVA) was computed with *group* as a between-subjects factor and participants' scores in the MET rhythmic subset as the dependent variable. Participants' scores in the cognitive tests, i.e., the MT and the BDS, their melodic aptitude as well as their weekly exposure to music (number of hours per week) were used as covariates. To ensure that the assumption of independence of the covariates (Miller and Chapman, 2001) was not violated, additional ANOVAs were conducted for each cognitive measure, i.e., BDS, MT, and melodic aptitude using *group* as a between-subjects factor. Along the same lines, a chi-square test was conducted to compare the three groups in terms of their weekly musical exposure.

## Results

Descriptive results and reliability tests are summarized in Table 1. In Table 2 language skills of the three L2 learner groups are shown.

**Table 1:** **Reliability tests and participants' score for each conducted task**.

**Tasks**	**Reliability (Cronbach's α)**	**German late L2 learners of English**		**Turkish early L2 learners of German**		**Turkish late L2 learners of German**	
		***M***	***SD***	***M***	***SD***	***M***	***SD***
Rhythmic aptitude test (MET subset)	0.627	64.50	8.30	70.15	9.76	71.78	8.17
Mottier Test	0.896	27.75	2.88	26.50	4.50	26.44	5.34
Backward digit span	0.694	8.53	2.79	7.73	1.88	7.18	2.30
Melodic aptitude test (MET subset)	0.821	64.78	12.07	65.75	11.79	67.02	10.78

**Table 2:** **Language skills of L2 learners**.

**Language skill (%)**	**German late learners of English**		**Turkish early learners of German**		**Turkish late learners of German**	
	***M***	***SD***	***M***	***SD***	***M***	***SD***
L1 Listening	99.67	1.79	94.81	10.51	99.25	2.66
L2 Listening	76.78	13.62	99.61	1.96	85.92	11.52
L1 Reading	99.67	1.79	85.38	20.63	99.25	2.66
L2 Reading	81.42	10.78	97.77	4.23	82.22	11.87
L1 Language independence	100	–	94.44	10.50	99.62	1.92
L2 Language independence	82.56	28.72	98.14	5.57	80.37	15.05

### Statistical analysis

Results of the ANOVA conducted with German L2 learners revealed no significant effect of the participants' English skills on their rhythmic performance, *F*_(2, 39)_ = 0.88, *p* > 0.1. An ANOVA investigating the independence of covariates as well as the chi-square test revealed that none of the covariates vary across *groups*, all *p*s > 0.05. For the rhythmic aptitude test, the conducted ANCOVA revealed a significant effect of *group*, *F*_(2, 78)_ = 9.29, *p* < 0.001, ω^2^ = 0.32.

Pairwise comparison of the adjusted means of participants' scores using the Holm's Sequential Bonferroni correction revealed a significant difference between German late L2 learners of English (*M* = 64.60, *SE* = 1.46) and Turkish late L2 learners of German (*M* = 71.78, *SE* = 1.57; *p* = 0.0002). In addition, German L2 learners' performance was significantly different from Turkish early L2 learners' (*M* = 70.15, *SE* = 1.91; *p* = 0.0023). A comparison between the two Turkish L2 learner groups did not yield significant differences (*p* = 0.40).

These findings are consistent with our initial hypothesis, namely despite controlling for individuals' cognitive abilities and melodic aptitude, group differences in rhythmic aptitude are confirmed.

**Figure 1:**
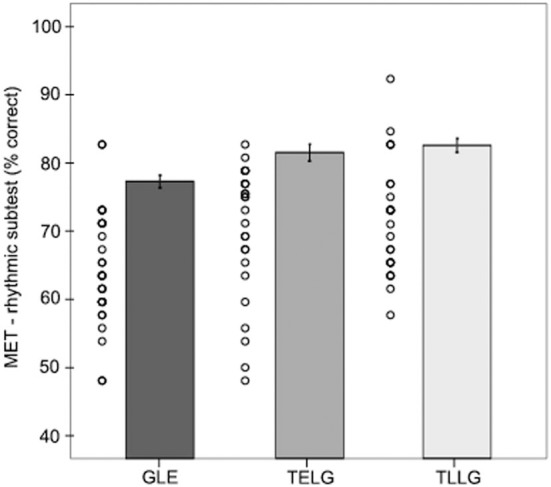
**Participants' and groups' performance in the rhythmic aptitude test (mean % correct).** Error bars indicate standard error.

Rhythmic performance of all participants (German late L2 learners of English, Turkish early and late L2 learners of German) in the MET rhythmic subset are depicted in Figure 1.

## Discussion

In the current study, we investigated the musical rhythm aptitude of Turkish early and late L2 learners of German and that of German late L2 learners of English to address two main issues. First, whether mastering languages with different rhythmic properties, such as Turkish and German, can enhance rhythm perception in music, and second, whether musical rhythm aptitude is modulated by L2 AoA.

Regarding the first question, results show that when controlling for participants' cognitive abilities i.e., STM and WM capacities, and for melodic aptitude, both Turkish L2 learner groups outperformed German L2 learners of English in terms of their rhythm aptitude.

Our findings suggest that specific linguistic properties, i.e., rhythmic information, may be transferred to the musical domain. This could be the case as individuals may recognize acoustic similarities in music and language, e.g., stress (Lerdahl and Jackendoff, 1983; Hayes, 1989; Jackendoff, 1989; Patel, 2003). This, in turn, may transfer from one domain to the other (Perkins and Salomon, 1989; Magne et al., 2003; Schön et al., 2004; Vuust et al., 2011).

Thus, being sensitive to different rhythmic properties as a result of mastering two languages may constitute a domain-specific ability, which results from domain-general skills (Perkins and Salomon, 1989; Salomon and Perkins, 1989), namely the ability to structure and organize events in time, i.e., rhythm (Jackendoff, 1989; Cummins and Port, 1998). This would parallel with recent findings in music research that a domain-specific skill enhances an individual's acoustic perception (Pantev et al., 2001; Kraus and Chandrasekaran, 2010; Vuust et al., 2012).

In addition, as rhythm is a valuable cue to discriminate between languages (Ramus, 2002; Nazzi and Ramus, 2003), perhaps L2 learners whose L1 is fundamentally different from their L2 with respect to rhythmic properties are more attentive and sensitive to acoustic variations than those L2 learners whose L1 and L2 rhythmic properties do not differ (e.g., German and English). This may lead to improved language recognition and selection. Given that Turkish and German are rather diverse concerning their rhythmic properties, rhythmic information may facilitate language selection and may allow cognitive resources to be allocated to other linguistic processes where they are most needed, such as speech segmentation.

Nevertheless, one may argue that our results could alternatively be explained by L2 learners' exposure to a different musical culture, namely Turkish music. In this sense, the higher level of rhythmic complexity found in Turkish music, such as the presence of a non-isochronous meter, so rare in Western music (Bates, 2010; Hannon et al., 2012), may contribute to higher rhythmic sensitivity among Turkish L2 learners. Thus, enhanced perception of rhythmic patterns could be influenced by the familiarity with a certain rhythm, and therefore, by a culture-specific listening experience (Hannon et al., 2012). Despite this reasoning, one should consider that the rhythmic variations participants were presented with can be found both in Western and Turkish music. Furthermore, rhythmic sentences varied with respect to one beat only, relativizing rhythmic complexity. As such, Turkish L2 learners of German should not start out with an advantage over German L2 learners of English in terms of musical rhythmic perception.

Additionally, one may think that our findings result from the nature of the Turkish language. This should not be the case, because Turkish and German present the same fundamental features establishing acoustic prominence in speech, i.e., duration and intensity creating lexical stress. Hence, it is unlikely that Turkish controls should have a rhythmic advantage over German controls in terms of their ability to discriminate these rhythmic properties. In addition, in a recent study Schmidt-Kassow et al. (2011b) reported that French native speakers detect stress variation in tonal sequences comparably to native speakers of German. Hence, their findings support the idea that no particular rhythmic class, i.e., stress-timing or syllable-timing, leads to an advantage in terms of rhythmic discrimination in a non-linguistic context. Nevertheless, in order to rule out the possibility that enhanced musical rhythmic perception may rely on the mastery of Turkish, Turkish monolingual controls should be further investigated.

Regarding the second issue addressed in this research, namely whether L2 AoA influences general rhythm perception, the current results indicate that musical rhythm perception does not seem to be subject to L2 AoA. The fact that both groups of Turkish L2 learners benefit from L1 and L2 rhythmic diversity seems to indicate that L2 speakers are sensitive and may learn, to some degree, L2 rhythmic properties beyond a sensitive period (Bailey et al., 1999; Goetry and Kolinsky, 2000; Field, 2003; Trofimovich and Baker, 2006).

This could be the case because the prominence created by rhythm is based on temporal acoustic perception, which can be learned and improved later on in life (Alain et al., 2007; Dahmen and King, 2007; Van Wassenhove and Nagarajan, 2007). Thus, speech rhythm could be less constrained by L2 AoA than other linguistic skills, such as complex syntactic processing and phonology (Johnson and Newport, 1989; Weber-Fox and Neville, 1996; Flege et al., 1999; Piske et al., 2001; Papadopoulou, 2005; Clahsen and Felser, 2006; Hernandez and Li, 2007).

In view of the current results, some questions remain. If enhanced musical rhythm aptitude found among L2 learners results from the selection of languages with distinct rhythmic properties, this could suggest that these L2 learners are also better in discriminating languages based on rhythmic information. Therefore, further investigations regarding language discrimination based on rhythmic properties should be carried out with L2 learners, whose L1 and L2 have different rhythmic properties.

Moreover, L2 learners from languages sharing some of their rhythmic properties, such as metric preference (e.g., German and Italian) or rhythmic organization (e.g., Spanish and French), should be tested. This could provide a more complete understanding of which rhythmic properties contribute more or less to an enhancement in musical rhythmic aptitude.

Such investigations should shed more light on if and how mastering languages with different rhythmic properties (e.g., stress position) may affect the ability to discriminate between languages, facilitating the selection of the target language and, therefore, speech processing.

## Conclusion

Our study is a first investigation on how distinct rhythmic properties in first and second languages may enhance musical rhythm aptitude. Results confirm an enhanced musical rhythm aptitude in Turkish early and late L2 learners of German compared to German late L2 learners of English. These findings should be taken as a starting point for future studies investigating the shared properties between language and music in the context of second language learning. Research into this specific topic will eventually provide a better understanding of how acoustic properties (e.g., sound duration and intensity) may be perceived and used across domains.

## Conflict of interest statement

The authors declare that the research was conducted in the absence of any commercial or financial relationships that could be construed as a potential conflict of interest.

## Footnotes

^1^Even though several studies refuted the idea of an objective isochrony (Lea, 1974; Beckman, 1982; Wenk and Wioland, 1982), on which the traditional rhythmic classification of languages is based (Pike, 1945; Abercrombie, 1967; Ladefoged, 1975) the terms “stress-timed,” “syllable-timed,” and “mora-timed” are still in use in the literature. For review and further discussion on this matter see (Patel, 2008).
