# Supplementary material for: Enhanced musical rhythmic perception in Turkish early and late learners of German
Source: Front Psychol. 2013 Sep 20;4:645. doi: 10.3389/fpsyg.2013.00645 (PMC3778315; doi:10.3389/fpsyg.2013.00645)
Supplement: Supplementary file 1 [file DataSheet1.PDF]

Self-reported language questionnaire

Part 1: Language profile

<table>
<tr><td colspan="2">Age:</td><td colspan="2">Gender: □ Feminine □ Masculine</td></tr>
<tr><td colspan="2">Country and place of birth:</td><td colspan="2"></td></tr>
<tr><td colspan="4">How long did/have you live/lived in your home country? (from ___ until _____):</td></tr>
<tr><td colspan="4">How long did you go to school or have any formal education in your home country?
(Please write down the name of the country, number of years and your age at that time):</td></tr>
<tr><td colspan="4">How long did you go to school or have any formal education in a foreign country? (Please write down the country, number of years and your age at the time):</td></tr>
<tr><td colspan="2">Profession/study path</td><td colspan="2">Years of formal training/ university</td></tr>
<tr><td colspan="4">Please estimate your language competence:

1 □ I speak and write only in my native language.
2 □ I speak and write in my native and in (an)other language(s).
3 □ I am bilingual: I speak fluently in my second language almost like a native speaker.
4 □ I am bilingual: I learned two languages simultaneously as a child.
5 □ I am multilingual: I speak fluently or like a native speaker more than one language and I master (an) additional language(s).</td></tr>
<tr><td colspan="4">Please write down the age at which you learned each language, describe briefly its learning situation (at home, in school, abroad) and write down your current use of each language.</td></tr>
<tr><td>Language:</td><td>Age:</td><td>Learning situation:</td><td>Current use</td></tr>
<tr><td>Native language</td><td></td><td></td><td></td></tr>
<tr><td>Second language</td><td></td><td></td><td></td></tr>
<tr><td>Other language
(please rank by competence)</td><td></td><td></td><td></td></tr>
</table>

Part 2: Language contact and self-assessment

1. In case you stayed in a country other than your home country longer than two weeks, please outline this briefly.

Country:	Length of stay/ Age:	Spoken language

2. Please write down all standardized language tests you took, including your score (also if you did not pass it).

Language:	Test:	Result:

3. Please estimate your language competence in your native, second and further languages.

I - Reading I find it …

1= very difficult to read and understand regular text (ex.: Newspaper), 10 = very easy to read and understand regular text.

a. Native language:

1 □	2 □	3 □	4 □	5 □	6 □	7 □	8 □	9 □	10 □

b. Second language:

1 □	2 □	3 □	4 □	5 □	6 □	7 □	8 □	9 □	10 □

c. Other language (which?)

1 □	2 □	3 □	4 □	5 □	6 □	7 □	8 □	9 □	10 □

II - Writing I find it…

1= very difficult to express ideas, 10 = very easy to express ideas.

a. Native language:

1 □	2 □	3 □	4 □	5 □	6 □	7 □	8 □	9 □	10 □

b. Second language:

1 □	2 □	3 □	4 □	5 □	6 □	7 □	8 □	9 □	10 □

c. Other language (which?)

1 □	2 □	3 □	4 □	5 □	6 □	7 □	8 □	9 □	10 □

III - Understanding I find it…

1= very difficult to understand spoken language (such as regular conversation, news), 10= very easy to understand spoken language.

a. Native language:

1 □	2 □	3 □	4 □	5 □	6 □	7 □	8 □	9 □	10 □

b. Second language:

1 □	2 □	3 □	4 □	5 □	6 □	7 □	8 □	9 □	10 □

c. Other language (which?)

1 □	2 □	3 □	4 □	5 □	6 □	7 □	8 □	9 □	10 □

IV - Speaking I find it…

1=very difficult to formulate and verbalize natural-sounding sentences in a conversation, 10= very easy to formulate and verbalize natural-sounding sentences in a conversation.

a. Native language:

1 □	2 □	3 □	4 □	5 □	6 □	7 □	8 □	9 □	10 □

b. Second language:

1 □	2 □	3 □	4 □	5 □	6 □	7 □	8 □	9 □	10 □

c. Other language (which?)

1 □	2 □	3 □	4 □	5 □	6 □	7 □	8 □	9 □	10 □

V - Grammar I find it…

1= very difficult to understand how a sentence is built, 10 = very easy to understand how a sentence is built.

a. Native language:

1 □	2 □	3 □	4 □	5 □	6 □	7 □	8 □	9 □	10 □

b. Second language:

1 □	2 □	3 □	4 □	5 □	6 □	7 □	8 □	9 □	10 □

c. Other language (which?)

1 □	2 □	3 □	4 □	5 □	6 □	7 □	8 □	9 □	10 □

VI - Language independence

1= If I use this language, I have to translate first in my native language, 10= I feel completely independent in this language and can “think” in it.

a. Native language:

1 □	2 □	3 □	4 □	5 □	6 □	7 □	8 □	9 □	10 □

b. Second language:

1 □	2 □	3 □	4 □	5 □	6 □	7 □	8 □	9 □	10 □

c. Other language (which?)

1 □	2 □	3 □	4 □	5 □	6 □	7 □	8 □	9 □	10 □

Part 3: Language preference and experience

Native language or first fluent language:	
Second language or second fluent language:	
Other language (which?)	

1. Which language do you use mostly on a daily basis?

□ Native language □ Second language

2. Which language did you learn when you learned how to speak?

□ Native language □ Second language □ both simultaneously

3. Please write down the native language of your parents and the language you spoke with each one of them.

	Native language:	Spoken language:
Mother:		
Father:		

4. If you learned two languages as a child, please describe briefly the learning situation in which this happened.

5. If you did not learn a second language as a child, please mark all the correct answers regarding your second language:

□ Traditional learning of a foreign language in school (number of years ___)
□ Learning a language in a school where the language is spoken (number of years_)
□ Contact through family members or through the partner (who ______________________)
□ Contact through an exchange program (where ____________and when________________)
□ Contact through a longer trip (where __________________and when_________________)
□ Contact via the profession or via the university (how_________________________________)
□ Other circumstances (which____)

6. Which language(s) do you speak more frequently at home with your family and friends? With whom do you speak these languages?

Spoken language at home: With whom:

7. In which environment was your native language used the most?

During your childhood*:*

□ home	□ school	□ both	□ other (which________________)

Currently*:*

□ home	□ work/university	□ both	□ other (which______________)

9. How much time in a day (in %) do you use each one of these languages (speaking, reading, writing and thinking)?

Native language __________ Second language _________ other language ________

10. (In) which language do you prefer to …

speak?

Native language __________ Second language _________ other language ________

hear?

Native language __________ Second language _________ other language ________

read?

Native language __________ Second language _________ other language ________

write?

Native language __________ Second language _________ other language ________

11. At which age did you begin to…

speak?

Native language ___________ Second language _________ other language ________

Understand spoken sentences?

Native language __________ Second language _________ other language ________

read?

Native language __________ Second language _________ other language ________

write?

Native language __________ Second language _________ other language ________

12. How often do you switch between your native and your second language and vice-versa?
1=I never switch languages, 10=I switch languages very often

1 □	2 □	3 □	4 □	5 □	6 □	7 □	8 □	9 □	10 □

13. How easy is it for you to switch between languages?
1=very difficult, 10=very easy

1 □	2 □	3 □	4 □	5 □	6 □	7 □	8 □	9 □	10 □

14. Please add any further information that you find relevant which was not captured by this questionnaire. THANK YOU!
